# Supplementary material for: ProteinShader: illustrative rendering of macromolecules
Source: BMC Struct Biol. 2009 Mar 30;9:19. doi: 10.1186/1472-6807-9-19 (PMC2672931; doi:10.1186/1472-6807-9-19)
Supplement: Additional file 1 — ProteinShader program without source code. This compressed file contains the complete ProteinShader program including associated libraries, but no source code. A README.txt file gives an overview of the ProteinShader distribution, and the index.html file in the help subdirectory has directions on getting started with the program as well as a set of tutorials. [file 1472-6807-9-19-S1.zip › ProteinShader-beta-0_9_4-binary/help/api/org/proteinshader/structure/exceptions/package-summary.html]

org.proteinshader.structure.exceptions (ProteinShader API)


|  |  |  |
| --- | --- | --- |
| **Overview** \| **Package** \| Class \| **Use** \| **Tree** \| **Deprecated** \| **Index** \| **Help** | |  |
| **PREV PACKAGE**   **NEXT PACKAGE** | **FRAMES**    **NO FRAMES**     **All Classes** |  |


---

## Package org.proteinshader.structure.exceptions

Holds the exception classes needed by org.proteinshader.structure and its
subpackages.

**See:**
  
          **Description**

| **Exception Summary** | |
| --- | --- |
| **BondLengthException** | Can be used to report that a Bond is unacceptably short or long. |
| **ColorOutOfRangeException** | Used to report that a color value is out of range. |
| **InvalidIDException** | Used to report that an ID is invalid or missing. |
| **InvalidRegionException** | Used to report that a Region (Helix or BetaStrand) is invalid because its start and end Residue IDs could not be matched to an existing sequence of Residues in a Chain. |
| **MissingAATypeException** | Used to report that an AminoAcid did not have a type specified. |
| **MissingAtomTypeException** | Used to report that an Atom did not have a type specified. |
| **MissingHetNameException** | Used to report that a heterogen name is missing. |
| **SegmentException** | Used to report an error while trying to create a Segment object. |

## Package org.proteinshader.structure.exceptions Description

Holds the exception classes needed by org.proteinshader.structure and its
subpackages.
